# Information Theory and an Entropic Approach to an Analysis of Fiscal Inequality

**DOI:** 10.3390/e21070643

**Published:** 2019-06-28

**Authors:** Duc Hong Vo

**Affiliations:** Business and Economics Research Group, Ho Chi Minh City Open University, Ho Chi Minh City 70000, Viet Nam; duc.vhong@ou.edu.vn

**Keywords:** entropy, dispersion, entropic approach, fiscal decentralisation, measurement, C02, H11, H29, H50, H77

## Abstract

In his influential study, Theil (1967) developed the notion of entropy on the basis of information theory. He then advocated the use of entropy-based measure for the analysis of income inequality. In this paper, the first of its kind, we apply Theil’s notion of entropy to public finances in multi-tiered governments, in particular for a measurement of fiscal decentralisation, which is currently very crude in terms of the ratio between local government revenue and total revenue. It is the claim of this paper that such an approach of measuring fiscal decentralisation completely ignores important distributional aspects of fiscal arrangements. Findings from this paper indicate that studies on measuring various aspects of fiscal activities—such as fiscal decentralisation—should carefully take into account the dispersion of revenue (and expenditure) across regions. On that basis, the entropic approach developed in this paper is able to accommodate these dispersions across subnational governments. As an illustration for the case of Vietnam, the true degree of fiscal decentralization has effectively been decreased in comparison with estimates from other simple measurements due to the presence of substantial dispersions of revenue and expenditure from the subnational governments across 63 provinces in Vietnam.

## 1. Introduction

In recent decades, fiscal decentralisation has become a central concern in countries around the world, especially in developing nations such as Argentina, Bolvia, Brazil, Colombia, Ethiopia, India, Mexico, and Nigeria; and countries in transition such as Bulgaria, China, Hungary, and the Russian Federation [1,2]. Fiscal arrangements among levels of government have been reformed in a manner that increases the extent to which subnational governments (SNGs) are assigned more expenditure and revenue-raising responsibilities. The theory of fiscal decentralization, both from expenditure and revenue assessment, has long been of interest among academics [3,4,5,6]. There is, however, a lack of a widely-accepted tool to measure the degree of fiscal decentralisation across countries. In previous studies, typically either revenue or expenditure from subnational governments is used without taking into account the fiscal autonomy of SNGs. For example, in his pioneering study, in 1972 Oates [7] used the national government’s share in total public revenue as the degree of fiscal centralisation. In 1998, Woller and Phillips [8] measured fiscal decentralisation in one of four ways: (i) the ratio of local government revenues to total government revenues; (ii) the ratio of local revenues less grants-in-aid to total government revenues; (iii) the ratio of local expenditures to total government expenditures; and (iv) the ratio of local government expenditures to total government expenditures less defence and social security expenditures. 

Similar measures of fiscal decentralisation can be found in previous studies [9,10,11,12,13], to name a few. None of these measures consider the autonomy of SNGs in their fiscal activities. In short, the linkage between “theory” and “measurement” is poor in much of the current literature.

Martinez-Vazquez et al. [14] argued that one crucial and yet unsolved issue in the empirical literature on decentralization is the proper measurement of decentralization itself. In one of the most recent papers on measuring fiscal decentralisation, Liu et al. [15] measured the degree of fiscal decentralization in the Chinese provinces by simultaneously considering expenditure decentralization and revenue decentralization. The authors argued that of these two indicators, expenditure decentralization, defined as the local share of total government expenditure has been widely used in previous empirical studies.

The above fiscal decentralisation indices have two potentially significant limitations. First, each subnational government (SNG) is implicitly treated as fiscally homogenous. In effect, per capita revenue and expenditure in each subnational region are implicitly assumed to be equal. However, SNGs typically involve large fiscal differences that may have implications for fiscal decentralisation. Second, but related to the first point, the structure of fiscal arrangements is ignored. SNGs are not differentiated by type—the state government level is not distinguished from the local government level. As such, the new indices developed earlier account only for the more fundamental influences on the fiscal autonomy and fiscal importance of SNGs, while ignoring the impact of fiscal differences between them. 

To redress these shortcomings, the background for the extension of the fiscal decentralisation index in future studies is developed, using information theory developed by Theil [16]. The main goals are to account for: (i) the distributions of state and local government revenue and expenditure shares between the regions physically defined by the border of state jurisdictions, and (ii) the distribution of state and local government revenue and expenditure shares within a physical region defined by the state-level governments. The concepts of “between-set entropy” and “within-set entropy” appear to have the potential to account for heterogeneity in fiscal shares across different levels of government.

The ideas of expected information of a direct message and an indirect message were originally developed by Theil in his influential book “Economics and Information Theory”. These ideas were further developed to measure the income inequality by comparing the income share with the population share of the states. These works lay a strong foundation for the development of an analytical framework of fiscal inequality which takes into account the dispersions of the revenue and expenditure of various levels of SNGs. This study is conducted to be devoted to this development.

The paper is structured as follows. Following this brief introduction, Section 2 discusses information theory including the concept of “entropy”. Section 3 of the paper presents the analytical framework for the analysis of subnational fiscal inequality. Decomposing revenue/expenditure inequality of a generic country is discussed at length in Section 4, followed by the C\conclusion in Section 5.

## 2. Information Theory

A possibility $E_{}$ will occur with the probability $x_{}$ with $0\;\leq \;x_{\;}\leq \;1$ where $x_{}=0$ means that this possibility will not be realised and $x_{\;}=\;1$ means that this possibility is definitely realised. When $x_{}$ is close to 0, say, $x_{}=0.01$, the information content of the message is very large. However, when $x_{}$ is close to one, say, $x_{}=0.95$, the message has provided little information content. To formalise these ideas, let h (x) be information content of a definite and reliable message x. It is obvious that h (x) will be the decreasing function of the probability x. This is because “the more unlikely the event before the message on its realisation, the larger the information content” [16]. Among many different decreasing functions, the logarithm of the reciprocal of the probability x is widely used.

(1) $$h\;(x)=\log\frac{1}{x}=-\;\log x$$

The other reason for the logarithmic function to be selected among many decreasing functions is the additivity of this function in the case of independent events. Suppose that $E_{1}$ with probability $x_{1}$ and $E_{2}$ with probability $x_{2}$ are stochastically independent, their product $x_{1}.x_{2}$ is the probability that both events occur. In this case, the information content of the message which informs us that “*both events did occur*”, $h(x_{1},\;x_{2})$, will be as follows:

(2) $$h(x_{1},x_{2})=\log\frac{1}{x_{1}.x_{2}}=\log\frac{1}{x_{1}}+\log\frac{1}{x_{2}}=h(x_{1})+h(x_{2})$$

The far right-hand side of the Equation (2) includes the information content of the message telling us that “Event $E_{1}$ occurred”, $h(x_{1})$, and the information content of the other message of “Event $E_{2}$ occurred”, $h(x_{2})$. As a consequence, as the Equation (2) shows, the information content of the message which informs us that “both events did occur” is the sum of the information content of “Event $E_{1}$ occurred” and the information content of “Event $E_{2}$ occurred”. This additivity is a very convenient property of definition in Equation (1).

### 2.1. The Entropy as the Information Content

In light of the previous discussion, it is clear that different values of probabilities $x_{i}$ of the event $E_{i}$ will provide different meanings. In short, it means that the lower the probability of an event occurring, the larger the “information content” of a message. 

Until the message is released, no one can predict how significant the “information content” will be as either $h\;(x_{1}),\;or\;h\;(x_{2}),\ldots ,\;or\;h\;(x_{n})$ with different probabilities $x_{1}\;\neq \;x_{2}\;\neq \;\ldots \;\neq \;x_{n}$ can occur. However, the average or expected information content can be calculated before the message arrives, since we know the probabilities. In this sense, the expected information content of the message is just the expected value of the information content, that is, the probability weighted average of $h(x_{1}),\;h(x_{2}),\ldots ,\;h(x_{n})$:

(3) $$H(x)=\sum_{i=1}^{n}x_{i}h(x_{i})=\sum_{i=1}^{n}x_{i}\log\frac{1}{x_{i}}=-\;\sum_{i=1}^{n}x_{i}\log x_{i}$$

Since $x_{i}$ is the probability for a particular event to occur, it follows that $0\;\leq \;x_{i\;}\leq \;1$ and $\log x_{i}$ will always be negative. As the product of $x_{i}\log x_{i}$ is always negative, $\sum_{i=1}^{n}x_{i}\log x_{i}\;<\;0$. Therefore, the negative of this sum, H(x), cannot be negative. In other words, H(x) cannot be negative since it is the weighted average, with all non-negative weights $x_{1},\;x_{2},\ldots ,\;x_{i}$, of the non-negative information values $h(x_{1}),\;h(x_{2}),\ldots ,\;h(x_{n})$. The measure H(x) is the expected information of a distribution, which Theil calls “entropy”. In addition, the value of the entropy H(x) has a lower limit of zero and the upper limit of logn, where n represents a number of events or possibilities, so that 0 ≤ H(x) ≤ logn.

### 2.2. The Appropriate Range for H(x)

The entropy H(x) falls in the range with a lower limit zero and the upper limit logn, where n represents a number of events or possibilities. 

For the lower limit, it is clear that when the event $E_{i}$ occurs with certainty, $x_{i}=1$, and $x_{j}=0\;\mathrm{for}\;\mathrm{all}\;i\;\neq \;j$. Thus, the probability vector $\left(x_{1},\;x_{2},\ldots ,\;x_{i},\ldots ,\;x_{n}\right)=(0,\;0,\ldots ,\;1,\ldots ,\;0)$. Then, $x_{i}\log x_{i}=0\;\mathrm{for}\;i=1,\ldots ,\;n\;\mathrm{and}$ $-\;\sum_{i=1}^{n}x_{i}\log x_{i}=0$. This establishes that the lower bound of H(x) is zero if and only if $x_{i}=1$ for some i.

Regarding the upper limit, the task now is to maximise the $\sum_{i=1}^{n}x_{i}\log x_{i}$, subject to $\sum_{i=1}^{n}x_{i}=1\;\mathrm{where}\;0\;\leq \;x_{i}\;\leq \;1$. To do this, we formulate the Lagrangian function:

$$L\;\left(x_{1},\ldots ,\;x_{n};\;\lambda \right)=-\sum_{i=1}^{n}x_{i}\log x_{i}-\lambda \left(\sum_{i=1}^{n}x_{i}\;-\;1\right)$$

where λ is the Lagrangian multiplier. The first-order condition is $\partial L/\partial x_{i}=-\;\log x_{i}-1-\lambda =0$. This is equivalent to $\log x_{i}=-\left(\lambda +1\right)$. This equation shows that $x_{i}$ is independent of i. This happens when and only when $x_{1}=x_{2}\;=\ldots =\;x_{i}=1/n$. When $x_{i}=1/n$, H(x) takes its upper value of logn. 

### 2.3. Entropy, Uncertainty and Dispersion

The measure H(x), defined in Equation (3), is known as the expected information content or the expectation of information. It is developed from the notion of the probability of occurrence of certain events. Based on the limits of this entropy, 0 ≤ H(x) ≤ logn, it is said that, prior to the presence of a message which states that A occurred, the more uncertainty there is, the larger the expected information content of the message. As a consequence, entropy H(x) can also be used to measure uncertainty of an event or an outcome. When an event is certain to occur, its probability is unity. There is no uncertainty, and H(x)=0, the lower limit, in this case. On the other hand, for a given number of events, uncertainty is at its maximum level when all events have the same probability, 1/n, of occurrence. This case corresponds with the upper limit of the expected information content H(x)=logn. Moreover, the level of uncertainty will increase with an increase in the number of outcomes n. For example, if there are only two possible outcomes, the probability of 1/2 for each outcome presents less uncertainty than in the case with 20 possible outcomes, which carries a probability of 1/20 to occur. In other words, the more equi-likely events that can occur, the more uncertainty there is.

In addition, the entropy H(x) can also be used to measure dispersion. The variance is the most common approach to measure dispersion of the distribution. The variance of a continuous random variable with a probability distribution f(x) is defined as: $\sigma_{}^{2}=\int_{-\;\infty }^{\infty }{\left(x\;-\;\mu \right)}^{2}f(x)dx$, where $\mu =\int_{-\;\infty }^{\infty }xf(x)dx$ is the mean. In the discrete case, entropy is defined as the negative value of the expected logarithms of event probabilities: $H(x)=-\;\sum_{i=1}^{n}x_{i}\log x_{i}$. When x is continuous, entropy is the negative value of expectation of the logarithms of the density: $H(x)=-\;\int_{-\;\infty }^{\infty }f(x)\log f(x)dx$. 

To illustrate, suppose x is normally distributed, with the mean μ and variance $\sigma^{2}$, so that:

f(x)=1σ2πe− 12(x − μ)2 σ2, so that $\log f(x)=-\;\log\sigma \sqrt{2\pi }-\frac{1}{2}\frac{{\left(x\;-\;\mu \right)}^{2}}{\sigma^{2}}$

The entropy now becomes:

$$\begin{array}{ll} H(x) & =\;-\;\int_{-\;\infty }^{\infty }f(x)\left\{-\;\log\sigma \sqrt{2\pi }-\;\frac{\left(1/2\right){\left(x-\mu \right)}^{2}}{\sigma^{2}}\right\}\;dx\\ & =\left(\log\sigma \sqrt{2\pi }\right)\int_{-\infty }^{\infty }f(x)dx+\frac{1}{2}\int_{-\infty }^{\infty }{\left(\frac{x-\mu }{\sigma }\right)}^{2}f(x)dx\;\\ & =\;\log\sigma \sqrt{2\pi }+\frac{1}{2}=\log\sigma +\left(\frac{1}{2}+\log\sqrt{2\pi }\right). \end{array}$$

Thus, the entropy of a normal distribution is the sum of the logarithm of the standard deviation σ and a constant equal to $1/2+\log\sqrt{2\pi }$. Since 1/2=(1/2)loge, the relationship between the entropy and the variance $\sigma^{2}$ of the normal distribution can also be expressed as: $H(x)=\left(1/2\right)\log\left(2\pi e\sigma^{2}\right)$. This shows that the entropy is an increasing function of the variance in the case of the normal distribution. Even though when things are not normally distributed however, the general idea that the entropy measures dispersion continues to hold. 

In conclusion, the entropy H(x) can be used to measure the expected information content, the uncertainty and the dispersion. The entropy H(x) is developed based on the concepts of probability alone, so it can take both numerical values (say, 0.1, 0.5,…) and “nominal” values (say, “rich” and “poor”). It sheds light on the view that the entropy H(x) is in contrast to the variance since the variance can only take the numerical values. 

#### An Indirect Message: Prior and Posterior Probabilities

When we take one possibility into consideration, an indirect message does not confirm any event but it does provide additional information regarding an event that may occur in the future. If so, then the expected information content will change. This is because, with the release of the message, some events have a higher chance of occurring and others have a lower probability of occurring, no guarantee of an event is provided with the release of the message. Similar to previous discussions, it is assumed we have n chances as $E_{1},\;E_{2},\ldots ,E_{n}$ with the probabilities to occur are $x_{1},\;x_{2},\ldots ,x_{n}$, respectively. These probabilities are known as prior probabilities since they existed before the message comes in. When the message comes in, these probabilities will be changed because with the presence of the message, some chances become more probable to occur and others become less probable to occur. The probabilities for these events $E_{1},\;E_{2},\ldots ,E_{n}$ to occur become $y_{1},\;y_{2},\ldots ,y_{n}$, respectively. These are called as *posterior* probabilities [16]. As a result, the sum of these posterior probabilities is unity. That means:

(4) $$\sum_{i=1}^{n}y_{i}=1,\;y_{i}\;\geq \;0\;\forall \;i=1,\;2,\ldots ,\;n.$$

These posterior probabilities are also non-negative. If it turns out that one of these probabilities is one, all the others are zero, then the message becomes a direct one since this message guarantees one particular event with probability of unity occurs. Recall from Equation (1) regarding the information content, we will then apply for the event $E_{i}$ to occur with the probabilities before and after the message is released (i.e., its prior and posterior probabilities) are $x_{i}$ and $y_{i}$, respectively. 

“Probability ex post” is the probability of the event to occur after the message is released. In this case, we will not know what happens for sure with the release of the message. In addition, the probability in this case is $y_{1}$. In addition, “probability ex ante” is the probability of the event to occur before the message is released, still $x_{i}$ in this case. Therefore, the information content in the case of an “indirect message” is as follows:

(5) $$h(y_{i},\;x_{i})=\log\frac{y_{i}}{x_{i}},$$

or in words: $\mathrm{The}\;\mathrm{information}\;\mathrm{received}\;\mathrm{with}\;\mathrm{message}=\log\left(\frac{\mathrm{probability}\;\mathrm{ex}\;\mathrm{post}}{\mathrm{probability}\;\mathrm{ex}\;\mathrm{ante}}\right).$

It is important to note that the message itself does not mention any possibility or event $E_{i}$ in particular. This means that the presence of the message does not guarantee the occurrence of any event. Any event has its own posterior probability $y_{i}$ to occur. In this case, the expected information of the indirect message is as follows:

(6) $$I(y:x)=\sum_{i=1}^{n}y_{i}\log\frac{y_{i}}{x_{i}}.$$

The expected information of an indirect message I(y:x) transforms the prior probabilities $x_{1},\;x_{2},\ldots ,\;x_{n}$ into the posterior probabilities $y_{1},\;y_{2},\ldots ,\;y_{n}$. And, I(y:x) is non-negative, which can be shown as follows. It is assumed that $y_{i}\;>\;0,\;i=1,\;2,\ldots ,\;n$ in the first instance. In addition, let us assume that, there exists a small number $\varepsilon_{i}\left(i=1,\;2,\ldots ,\;n\right),$ such that $\sum_{i=1}^{n}y_{i}\varepsilon_{i}=0.$ In this case, the equation $x_{i}=y_{i}(1+\varepsilon_{i})$ holds, or equivalently: $x_{i}/y_{i}=1+\varepsilon_{i}$. Equation (6) can then be rewritten as follows:

(7) $$I(y:x)=-\sum_{i=1}^{n}y_{i}\log\;(1+\varepsilon_{i}).$$

**Figure 1:**
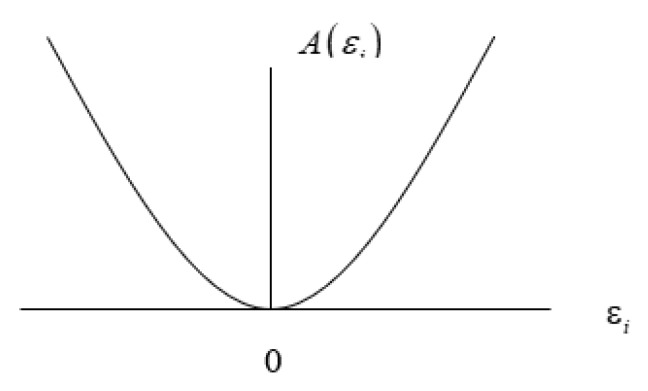
The expected information content of an indirect message.

Since $\sum_{i=1}^{n}y_{i}\varepsilon_{i}=0$, Equation (7) can be rewritten as $I(y:x)=\sum_{i=1}^{n}y_{i}\left[\varepsilon_{i}\;-\;\log\;(1+\varepsilon_{i})\right]$. In proving that I(y:x) is non-negative, because $y_{i}\geq \;0$, it is only necessary to prove that $A\left(\varepsilon_{i}\right)=\left[\varepsilon_{i}\;-\;\log\;(1+\varepsilon_{i})\right]\;\geq \;0$. Taking the first-order derivative of $A\left(\varepsilon_{i}\right)$ is: $dA/d\varepsilon_{i}=1-1/\left(1+\varepsilon_{i}\right)=\varepsilon_{i}/\left(1+\varepsilon_{i}\right)$. This is obvious that this derivative disappears when $\varepsilon_{i}=0$. In addition, the derivative $dA/d\varepsilon_{i}$ is positive when $\varepsilon_{i}\;>\;0$ and negative when $\varepsilon_{i}\;<\;0$. However, regardless of the value of $\varepsilon_{i}$, negative or positive, the function $A\left(\varepsilon_{i}\right)$ is always positive as long as $\varepsilon_{i}$ is a small number. It is clear that $A\left(\varepsilon_{i}\right)=0$ when $\varepsilon_{i}=0$, and $A\left(\varepsilon_{i}\right)\;>\;0$ when $\varepsilon_{i}\;\neq \;0$, and the function looks like below. In short, as presented in Figure 1 below, the function $A\left(\varepsilon_{i}\right)\;\geq \;0,$ so that I(y:x) ≥ 0 and the equality sign holds when and only when each $\varepsilon_{i}$ disappears, that is, when $x_{i}=y_{i}$ for all i. It means that the expected information of an indirect message disappears when all probabilities are left unchanged.

It is important to further note that, as previously discussed, in the case where $x_{1}=x_{2}=\ldots =x_{n}=1/n$, the entropy is at its maximum value. That is H(x)=logn. In this case, the expected information content of an indirect message I(y:x) is:

(8) $$\log n-\sum_{i=1}^{n}y_{i}\log\frac{1}{y_{i}}=\log n-H(y).$$

Equation (8) tells us that, in a special case for equal prior probabilities, the expected information of an indirect message is the difference between the maximum value (logn) of the entropy of the posterior probabilities, and the actual value of the entropy H(y).

In addition, the expected information of an indirect message I(y:x) as in Equation (7) can be named as the information inaccuracy. This is because the message transforms the prior probabilities (before a realisation of an event) into posterior probabilities (after a realisation of an event). The presence of the posterior probabilities reveals how allocation of occurrence among events actually took place. When the message has a zero expected information (i.e., I(y:x)=0), we have $x_{i}=y_{i},\;\mathrm{where}\;i=1,2,\ldots ,n.$ In this case, the forecast is perfect. As a result, the higher the expected information of an indirect message is, the more inaccurate the forecast is. 

### 2.4. The Expected Information Content

The following section explores the link between the expected information content of an indirect message with both prior and posterior probabilities, being weighted by respective posterior probabilities. Since the sums of prior or posterior probabilities are both unity, the expected information content of an indirect message could be expressed as the weighted sum of these two probabilities. From Equation (6), the expected information content of an indirect message is the sum of n terms involving $x_{i}$ and $y_{i}$. The $x_{i}$ and $y_{i}$ are prior and posterior probabilities of an event $E_{i}$ to occur and $\sum_{i=1}^{n}x_{i}=\sum_{i=1}^{n}y_{i}=1$. Suppose that $y_{i}\;>\;x_{i}$ for each i, so that $y_{i}-x_{i}\;>0$ for each i. This is contrary to the fact that the sum of both sets of probabilities is unity. As a result, n terms in Equation (6) must consist of some negative terms and some positive terms so that $y_{i}\;>\;x_{i}$ for some i and $y_{j}\;<\;x_{j}$ for some j where i ≠ j. We start with the function in logarithms $\log\left(y_{i}/x_{i}\right)$ which we express as:

(9) $$\log\frac{y_{i}}{x_{i}}=-\;\log\left[1+\frac{x_{i\;}-\;y_{i}}{y_{i}}\right].$$

For convenience, let $a=\left(x_{i}\;-\;y_{i}\right)/y_{i}$, so that we can write Equation (9) as $\log\left(y_{i}/x_{i}\right)=-\;\log\left(1+a\right).$ Function f(a) can be expanded as Maclaurin series:

(10) $$f(a)=f(0)+\frac{f^{\prime }(0)}{1!}a+\frac{f^{\prime \prime }(0)}{2!}\;a^{2}+\;\frac{f^{\prime \prime \prime }(0)}{3!}\;a^{3}+\frac{f^{4}(0)}{4!}\;a^{4}\;+\ldots \;$$

With f(a)=− log(1+a), and $a=\left(x_{i}\;-\;y_{i}\right)/y_{i},$ we have f(0)=0, $f^{\prime }\left(0\right)=-\;1$, $f^{\prime \prime }\left(0\right)=1$, $f^{\prime \prime \prime }\left(0\right)=-\;2$ and $f^{4}\left(0\right)=6.$ Using these values in Equation (9), we then obtain:

(11) $$-\;\log\left(1+\frac{x_{i\;}-\;y_{i}}{y_{i}}\right)=-\;\frac{x_{i\;}-\;y_{i}}{y_{i}}+\frac{1}{2}\;{\left(\frac{x_{i\;}-\;y_{i}}{y_{i}}\right)}^{2}-\;\frac{1}{3}\;{\left(\frac{x_{i\;}-\;y_{i}}{y_{i}}\right)}^{3}+\;\frac{1}{4}{\left(\frac{x_{i\;}-\;y_{i}}{y_{i}}\right)}^{4}-\;\ldots$$

The above expansion converges if $\left(x_{i}\;-\;y_{i}\right)/y_{i}<\;1,$ or $x_{i}\;<\;2y_{i}$. The first term of the right-hand side of Equation (11) is worth considering. If we multiply it by $y_{i}$ and take the sum, we have: $-\;\sum_{i=1}^{n}y_{i}\left[\left(x_{i\;}-\;y_{i}\right)/y_{i}\right]=-\;\sum_{i=1}^{n}\left(x_{i\;}-\;y_{i}\right)=0.$ The expected information content now becomes:

(12) $$I(y:x)=\sum_{i=1}^{n}y_{i}\log\frac{y_{i}}{x_{i}}=\frac{1}{2}\;\sum_{i=1}^{n}\frac{{\left(x_{i}-y_{i}\right)}^{2}}{y_{i}}-\frac{1}{3}\;\sum_{i=1}^{n}\frac{{\left(x_{i}-y_{i}\right)}^{3}}{y_{i}{}^{2}}+\frac{1}{4}\;\sum_{i=1}^{n}\frac{{\left(x_{i}-y_{i}\right)}^{4}}{y_{i}{}^{3}}-\ldots$$

From these results, the expected information content of an indirect message can also be used to represent information inaccuracy because it translates the prior probability into posterior probability: the higher the differences between these two probabilities $x_{i}-y_{i}$ are, the more inaccurate the information is. 

Vo [14] represented that many previous attempts to measure the degree of fiscal decentralisation involve the use of some form of share of revenue/expenditure at lower-level jurisdictions in the national total. It is the claim of this paper that such an approach completely ignores important distributional aspects of fiscal arrangements. Consider two hypothetical economies, A and B. In both economies, government spending and revenue at the national level accounts for 50 percent of the total, so that the remaining 50 percent is the responsibility of SNGs. In country A, there are only two large subnational governments, each with an equal share of total subnational fiscal activity (i.e., 50 percent each); while in country B there are 100 subnational units, each accounting for 1 percent of the 50 percent total. It is clear that there is substantially more fiscal decentralisation in B as compared to A. However, an exclusive focus of the split of the total between the national and subnational levels would lead one to erroneously conclude that both economies exhibit the same degree of fiscal decentralisation. In other words, both the first and second moments of the distribution of revenue/expenditure are important for understanding the workings of fiscal arrangements. 

## 3. An Analytical Framework for the Analysis of Subnational Fiscal Inequality

In his influential study, Theil [16] advocated the use of entropy-based measure for the analysis of income inequality. In this section, we apply Theil’s notion of entropy to public finances in multi-tiered governments. The analysis that follows is devoted to the development of an analytical framework which reveals SNGs’ fiscal inequality in term of revenue shares among SNGs. The same framework can be directly applied to the expenditure shares among SNGs. The notion of fiscal inequality (or fiscal dispersion) is important for fiscal theory on decentralisation because it accounts for the heterogeneity of various subnational units in terms of revenue and expenditure shares. However, it should be emphasised that fiscal inequality and fiscal equalisation are two distinct concepts, in that fiscal equalisation is not designed to redress the notion of fiscal inequality in this paper. Specifically, the concept of fiscal inequality in this paper relies on “money” (such as revenue and expenditure of subnational governments) as the unit of comparison, whereas, the fiscal equalisation process (such as that adopted in Australia) is concerned with equalising the capacity of SNGs to provide the same “real” level of service.

It is assumed that a country has P states (the second level of government) and Q local councils (the third level of government) and each local council belongs to one state. Let N=P+Q be the total number of local and state governments, the number of subnational governments (SNGs). It is further assumed that each subnational government accounts for a non-negative fraction of total subnational revenue, to be denoted by $r_{i}$ which, for short, we shall refer to as the “regional revenue share”. The sum of these all revenue shares is equal to unity: $\sum_{i=1}^{N}r_{i}=\;1,\;r_{i}\;\geq \;0\;\forall \;i=1,\;\ldots ,\;N.$ Let r denote the vector of revenue shares $r_{1},\;\ldots ,\;r_{N}$. The entropy of revenue shares is defined as:

(13) $$H(r)=\sum_{i=1}^{N}r_{i}\;\log\frac{1}{r_{i}}.$$

Entropy H(r) can be regarded as the measure of the equality with which revenue is distributed among the SNGs. When the revenue distribution is extremely equal in that each SNG has the same revenue share (i.e., $r_{i}=1/N$) and revenue entropy is at its maximum: H(r)=logN. At the other extreme, when only one SNG collects all SNGs revenue so that others have no revenue at all (i.e., $r_{i}=1$ and $r_{j}=0$ for i ≠ j), the minimum value of the entropy is achieved: H(r)=0. As a result, the range of the entropy is 0 ≤ H(r) ≤ logN.

In the context of considering the relevance of the distribution of revenue among SNGs for its impact on fiscal decentralisation, it is appropriate to focus on revenue inequality between SNGs, rather than revenue equality. Revenue inequality is measured by deducting the revenue entropy, H(r), from its maximum value, logN:

(14) $$\log N\;-\;H(r)=\log N\;-\;\sum_{i=1}^{N}r_{i}\log\frac{1}{r_{i}}=\sum_{i=1}^{N}r_{i}\log Nr_{i}.$$

Due to the constraints on the range of the entropy H(r), it is clear that the range of this measure of revenue inequality is 0 - perfect equality (when H(r)=logN) - and logN - maximum inequality (when H(r)=0). The entropy H(r) is an attractive way to measure equality as it satisfies three axioms or tests described below. 

Axiom 1: The proportionality test

The entropy in Equation (13) is expressed in terms of the revenue shares of SNGs. Thus, if all revenues change proportionally, the shares do not change, and measure in euqation (14) remains unchanged. This invariance of revenue inequality to a proportional change is the proportionality test. 

Axiom 2: The “Haves and Have Nots” test

The upper limit of H(r) increases with N, so the maximum value of the inequality measure in Equation (14) rises with N. Consider two hypothetical countries. First, in a two-subnational region country, there is perfect inequality when one SNG accounts for all revenue, and the other has no revenue. The entropy of the revenue shares is zero, and the value shown in Equation (13) is log2. Second, in a society consisting of 10,000 SNGs, revenue inequality is at maximum when 9,999 SNGs have no revenue. The value of revenue inequality is now log10,000. It is obvious that revenue distribution in the latter is much more unequal than the first country. In the first country, one-half of the SNGs (one SNG) accounts for all subnational revenue and the other half has no revenue. As a result, revenue inequality of the second country is as unequal as for the first country when one-half of the SNGs account for all subnational revenue and when each of these has the same revenue. The concern is that whether revenue inequality, as expressed in Equation (13), satisfies this condition. The following material reveals that this is true by showing that as a larger fraction of SNGs join the “revenue” group, revenue inequality falls. This establishes that revenue inequality will be uniquely determined by the size of the revenue group (which we call “the haves”) relative to the “no-revenue” group (“the have nots”).

Assume there is a set S which consists of M subnational governments where 0 < M ≤ N. It is further assumed that SNGs in set S account for all subnational revenue, so that SNGs outside set S have no revenue. Also, within set S, each SNG accounts for the same amount of revenue (i.e., for $i\;\in \;S,\;r_{i}=\;1/M.\;$). The inequality measure in Equation (13) then becomes:

$$\sum_{i=1}^{N}r_{i}\log Nr_{i}=\sum_{i\;\in \;S_{i}}r_{i}\log Nr_{i}=\frac{1}{M}\log N\frac{1}{M}+\frac{1}{M}\log N\frac{1}{M}+\ldots ,$$

or:

(15) $$\sum_{i=1}^{N}r_{i}\log Nr_{i}=\log\frac{N}{M}=\log\frac{1}{\theta }\;,$$

where θ=M/N is the fraction of SNGs in the country who jointly account for all subnational revenue. The application of the last member of Equation (15) to the second example above with N=10,000 and θ=5000/10,000 =1/2, reveals that revenue inequality is also log2.

When revenue is equally distributed among some groups of SNGs in the society, and the remaining SNGs outside these groups have no revenue, revenue inequality of the country is determined solely by the fraction—the ratio of the number of SNGs in the group to the total number of SNGs. In both examples above, this ratio is 1/2, and the revenue inequality is log2. This result is in consistence with intuition: when the number of SNGs receiving revenue, M, increases, revenue distribution becomes more equal. The above discussion shows that as the inequality (3.3) decreases as the share of a number of SNGs which receive revenue rises, this measure satisfies the “Haves and Have Nots” axiom.

Axiom 3: The revenue transfer test

Consider an economy consisting two SNGs only A (rich) and B (poor) with the revenue shares $r_{A}$ and $r_{B}$, where $r_{A}\;>\;r_{B}.$ Suppose that some revenue is transferred from A to B, such that $dr_{A}+dr_{B}=0.$ A reasonable measure of revenue inequality should indicate that such a transfer from the rich SNG to the poor SNG has the effect of decreasing inequality. Does Equation (13) satisfy this property? The following material shows that it does have this property.

It is assumed that there are G sets of SNGs, to be denoted by $S_{1}\text{​},\;\ldots ,S_{G},$ and each SNG belongs to one and only one set. Let $N_{g}$ be a number of SNGs in set $S_{g}$, with $\sum_{g=1}^{G}N_{g}=\;N.$ To give some practical significance to the symbols, consider a three-tiered government: tier 1—national government; tier 2—state government; and tier 3—local government. $S_{g}$ represents the set of state and local governments in the geographical region defined by the jurisdiction of State g. $N_{g}$ is the total number of state and local governments within the jurisdiction defined by State g. In view of this, the entropy of revenue shares, Equation (13), can now be expressed as:

(16) $$H(r)=\sum_{g=1}^{G}\left[\sum_{i\;\in \;S_{g}}^{}r_{i}\log\frac{1}{r_{i}}\right],$$

where the component inside the square brackets is the entropy of revenue shares within set $S_{g}.$ Let $R_{g}$ be the sum of revenue shares of all SNGs in set $S_{g},$ $R_{g}=\sum_{i\;\in \;S_{g}}r_{i};$ this $R_{g}$ is the revenue share of group g with $\sum_{g=1}^{G}R_{g}=1.$ The entropy of revenue shares within set $S_{g}$ can be expressed as:

$$\begin{array}{ll} \sum_{i\;\in \;S_{g}}^{}r_{i}\log\frac{1}{r_{i}} & =\;R_{g}\;\left[\sum_{i\;\in \;S_{g}}^{}\frac{r_{i}}{R_{g}}\;\left(\log\frac{1}{r_{i}/R_{g}}\;\times \;\frac{1}{R_{g}}\right)\right]\;\\ & =\;R_{g}\;\sum_{i\;\in \;S_{g}}^{}\frac{r_{i}}{R_{g}}\;\log\frac{1}{r_{i}/R_{g}}\;+\;R_{g}\log\frac{1}{R_{g}}. \end{array}$$

Thus, if we define $H_{g}\left(r_{g}\right)\;=\sum_{i\;\in \;S_{g}}^{}\frac{r_{i}}{R_{g}}\;\log\frac{1}{r_{i}/R_{g}},$ where $r_{g}$ is the vector of $r_{i}$ that fall under $S_{g},$ as the within-set entropy, we have:

(17) $$\sum_{i\;\in \;S_{g}}^{}r_{i}\log\frac{1}{r_{i}}=\;R_{g}H_{g}\left(r_{g}\right)+R_{g}\log\frac{1}{R_{g}}.$$

Combining Equations (16) and (17), the total entropy becomes:

(18) $$H(r)=\sum_{g=1}^{G}R_{g}H_{g}\left(r_{g}\right)\;+\sum_{g=1}^{G}R_{g}\log\frac{1}{R_{g}}.$$

On the right-hand side of this equation, the first component is a weighted average of the within-set entropies $H_{1}\left(r_{1}\right),\ldots ,\;H_{G}\left(r_{G}\right),$ with the group revenue shares $R_{1},\ldots ,\;R_{G}$ as the weights. The second term on the right of Equation (18) is the between-set entropy, $\sum_{g=1}^{G}R_{g}\log\left(1/R_{g}\right).$

Let consider the N > 2 case where there are three groups of SNGs: (i) Group A with only one SNG A; group B with SNG B; and (iii) group C with (N − 2) SNGs comprising every SNG in the economy except A and B. These three groups are denoted by $S_{A},\;S_{B},\;\mathrm{and}\;S_{C}.$ We assume that the joint revenue share of A and B is a constant, i.e., $r_{A}+r_{B}=R_{A\;+\;B}=\mathrm{constant}.$ This implies that the revenue share of group C, $R_{C},$ is also constant at $1\;-\;R_{A\;+\;B}.$ It is further assumed that there are no revenue transfers to or from the other SNGs of the society in $S_{C}.$ We now apply decomposition of Equation (18) to this economy. The weighted average of the within-group entropies, the first term on the right-hand side of Equation (18), is:

(19) $$\sum_{g=1}^{G}R_{g}H_{g}\left(r_{g}\right)=R_{A}H_{A}\left(r_{A}\right)+R_{B}H_{B}\left(r_{B}\right)+R_{C}H_{C}\left(r_{C}\right)=R_{C}H_{C}\left(r_{C}\right).$$

where $H_{C}\left(r_{C}\right)\;=\sum_{i\;\in \;S_{C}}^{}\frac{r_{i}}{R_{C}}\;\log\frac{1}{r_{i}/R_{C}},$ with $r_{C}$ is the vector of $r_{i}$ that fall under group $S_{C},$ is the within-group entropy of group C. The first and second components in Equation (19), the within-group entropies for groups A and B, disappear because there is only one SNG in each group. In addition, the between-group entropy, the second term on the right-hand side of Equation (18), now becomes:

(20) $$\sum_{g=1}^{G}R_{g}\log\frac{1}{R_{g}}=R_{A}\log\frac{1}{R_{A}}+R_{B}\log\frac{1}{R_{B}}+R_{C}\log\frac{1}{R_{C}}.$$

Substituting Equations (19) and (20) into Equation (18), the total entropy for this three-group country becomes:

(21) $$H\left(r\right)=R_{A}\log\frac{1}{R_{A}}+R_{B}\log\frac{1}{R_{B}}+R_{C}\log\frac{1}{R_{C}}+R_{C}H_{C}\left(r_{C}\right).$$

When we transfer revenue from A to B, with the distribution within $S_{C}$ remaining unchanged, Equation (21) can be expressed as:

(22) $$H\left(r\right)=R_{A}\log\frac{1}{R_{A}}+R_{B}\log\frac{1}{R_{B}}+\mathrm{constant}.$$

The constant in Equation (22) includes $R_{C}\log\left(1/R_{C}\right)$ and $R_{C}H_{C}\left(r_{C}\right).$ In words, the total entropy of the three-group country is equal to the total entropy of two-group country plus a constant. Accordingly, the impact on inequality of a transfer from A to B is the same in the N > 2 case as it is in the N=2 case. 

To summarise this discussion, revenue inequality decreases if there is a transfer of revenue from the rich SNG to the poor SNG. This conclusion holds for a society with two-subnational regions (N = 2), as well as in the higher-dimensional case (N > 2). In short, it is clear that the measure of revenue inequality satisfies the revenue transfer test.

## 4. Decomposing Revenue/Expenditure Inequality

In the above, we decomposed revenue equality into within-set and between-set terms. We now show that revenue inequality can be similarly decomposed. 

Recall from Equation (18) that the entropy is decomposed into two distinct components: a weighted average of the within-set entropy and the between-set entropy. Furthermore, as in Equation (14), inequality is measured by the difference between the maximum value of the entropy, logN and the entropy H(r). Thus, by combining Equations (14) and (18), revenue inequality can be expressed as:

(23) $$\log N\;-\;H(r)=\log N\;-\;\sum_{g=1}^{G}R_{g}H_{g}(r_{g})\;-\sum_{g=1}^{G}R_{g}\log\frac{1}{R_{g}}.$$

The right-hand side of Equation (23) remains unchanged if we subtract and add $\sum_{g=1}^{G}R_{g}\log N_{g},$ where $R_{g}$ and $N_{g}$ are the revenue share of and a number of SNGs in set $S_{g},$ respectively:

$$\begin{array}{ll} \log N\;-\;H(r) & =\;\sum_{g\;=\;1}^{G}R_{g}\left(\log N_{g}\;-\;H_{g}(r_{g})\right)\;+\;\log N\;-\;\sum_{g\;=\;1}^{G}R_{g}\log\frac{N_{g}}{R_{g}}\\ & =\;\sum_{g\;=\;1}^{G}R_{g}\left(\log N_{g}\;-\;\sum_{i\;\in \;S_{g}}^{}\frac{r_{i}}{R_{g}}\;\log\frac{1}{r_{i}/R_{g}}\right)\;+\;\sum_{g\;=\;1}^{G}R_{g}\log\frac{R_{g}}{N_{g}/N}. \end{array}$$

As the result, revenue inequality can be expressed as follows:

(24) $$\log N\;-\;H(r)=\sum_{g=1}^{G}R_{g}\left[\sum_{i\;\in \;S_{g}}^{}\frac{r_{i}}{R_{g}}\log\frac{r_{i}/R_{g}}{1/N_{g}}\right]\;+\sum_{g=1}^{G}R_{g}\log\frac{R_{g}}{N_{g}/N}.$$

Equation (24) reveals that revenue inequality consists of two distinct components: (i) a weighted average of within-set inequalities and (ii) a between-set inequality. The right-hand side of Equation (24) parallels the decompositions given by Equation (18). The meaning of the two components of Equation (24) is discussed further in what follows.

### 4.1. The within-Set Inequalities

The first component on the right-hand side of Equation (24) is a weighted average of the within-set inequalities:

(25) $$\sum_{g=1}^{G}R_{g}\left[\sum_{i\;\in \;S_{g}}^{}\frac{r_{i}}{R_{g}}\log\frac{r_{i}/R_{g}}{1/N_{g}}\right].$$

The term $r_{i}/R_{g}$ is the conditional revenue share of SNG i within group $S_{g},$ that is, SNG i’s revenue share within the group. Also, $N_{g}$ represents a number of SNGs in group $S_{g}.$ Equation (25) comprises two weighted averages: (a) $Z_{g}=\sum_{i\;\in \;S_{g}}^{}\frac{r_{i}}{R_{g}}\log\frac{r_{i}/R_{g}}{1/N_{g}},$ the within-set revenue inequality for group $S_{g},$ and (b) $\sum_{g=1}^{G}R_{g}Z_{g},$ the weighted average of the within-set revenue inequalities. We discuss each in turn.

If each SNG in set $S_{g}$ receives an equal revenue share, then $r_{i}/R_{g}=k$ (say). However, as $\sum_{i\;\in \;S_{g}}^{}\left(r_{i}/R_{g}\right)=1,$ it follows that $k=1/N_{g}.$ When each SNG has an equal share of the group’s revenue, i.e., $r_{i}/R_{g}=\;1/N_{g},\;i\;\in \;S_{g},$ then there is no dispersion of the revenue distribution within the group, the perfect equality. Accordingly, the extent to which the $N_{g}$ ratios deviate from unity is a measure of revenue inequality within set $S_{g}.$

(26) $$\frac{r_{i}/R_{g}}{1/N_{g}},\;i=1,\ldots ,\;N_{g}$$

The within-set measure of revenue inequality, the term in square brackets of Equation (25), is a weighted average of the logarithms of the ratios in Equation (26), the weights being the conditional revenue shares.

### 4.2. The between-Set Inequality

The second term on the right-hand side of Equation (24) is the between-set inequality:

(27) $$\sum_{g=1}^{G}R_{g}\log\frac{R_{g}}{N_{g}/N}.$$

The basic ingredient of inequality from Equation (27) is the contrast between two sets of shares, the revenue shares of the G groups, $R_{1},\ldots ,\;R_{G}$ and the corresponding population shares, $N_{1}/N,\ldots ,\;N_{G}/N.$ If all groups receive their pro-rata shares of revenue based on population, i.e., $R_{g}=N_{g}/N,\;g=1,\ldots ,\;G,$ then there is no dispersion of revenue distribution and we have perfect between-set revenue equality. 

In summary, total inequality consists of two components: the weighted average of the within-set inequality and the between-set inequality. Interestingly, it is clear that both components are of the form of the expected information content of an indirect message which was previously discussed in Section 2. For the within-set inequality, the prior and posterior probabilities are $1/N_{g}$ and $r_{i}/R_{g}$, respectively. Similarly, for a between-set inequality, $N_{g}/N$ and $R_{g}$ are prior and posterior probabilities. Furthermore, from Equation (24), the revenue inequality, can be written as:

(28) $$\log N-H(r)=\sum_{i=1}^{N}r_{i}\log Nr_{i}=\sum_{i=1}^{N}r_{i}\log\frac{r_{i}}{1/N}.$$

The far right-hand side of Equation (28) reveals that total revenue inequality can also be expressed in the form of the expected information content of an indirect message. In this case, the prior and posterior probabilities are 1/N and $r_{i},$ respectively. With this perspective, it is clear that the message that transforms the vector ${\left[1/N,\ldots ,\;1/N\right]}^{\prime }$ into ${\left[r_{1},\ldots ,\;r_{N}\right]}^{\prime }$ is equivalent to two sub-messages. The first message transforms ${\left[1/N_{g},\ldots ,\;1/N_{g}\right]}^{\prime }$ into ${\left[r_{1}/R_{g},\ldots ,\;r_{g}/R_{g}\right]}^{\prime },$ g=1,…, G, which could be called “the within-set message”, and the second message transforms ${\left[N_{1}/N,\ldots ,\;N_{G}/N\right]}^{\prime }$ into ${\left[R_{1},\ldots ,\;R_{G}\right]}^{\prime },$ which is “the between-set message”.

The entropic analysis of fiscal arrangements can, of course, be extended to the expenditure shares of SNGs in exactly the same manner as applied above to revenue shares.

### 4.3. A Note on Notation

In the above discussion, the results are formulated in logarithmic terms. For future reference, it is convenient to take the antilogarithm of the inequality measure. 

Recall the second component on the right-hand side of Equation (24), the between-set inequality, which is a weighted average of the logarithms of the ratios of the set revenue shares and the corresponding institutional shares, $\sum_{g=1}^{G}R_{g}\log\frac{R_{g}}{N_{g}/N}.$ Let $m_{i}$ and $q_{i}$ be the revenue share and institutional share of the *i*th region, that is, $m_{i}=M_{i}/M,$ where $M_{i},\;M$ are the revenue of the *i*th region and the total economy, and $q_{i}=Q_{i}/Q,$ where $Q_{i},\;Q$ are the number of SNGs in the *i*th region and the total number of SNGs in the economy. As a result, $\frac{m_{i}}{q_{i}}=\frac{M_{i}/M}{Q_{i}/Q}=\frac{M_{i}/Q_{i}}{M/Q}.$ The numerator of this ratio is revenue per SNG of the *i*th region, while the denominator is revenue per SNG. If $m={\left[m_{1},\ldots ,\;m_{N}\right]}^{\prime }$ and $q={\left[q_{1},\ldots ,\;q_{N}\right]}^{\prime },$ the between-region inequality can be expressed in terms of information theory as:

$$I\left(m:q\right)=\sum_{i=1}^{N}m_{i}\log\frac{m_{i}}{q_{i}}.$$

The ratio $m_{i}/q_{i}$ is “deflated” per SNG revenue of the *i*th set. The term “deflated” here means that revenue is expressed as relative to national revenue for SNG. The above I(m:q) is the logarithm of a weighted average of deflated revenue per SNG, so that the corresponding geometric mean is:

(29) $$e^{I\left(m:q\right)}={\prod_{i=1}^{N}\left(\frac{m_{i}}{q_{i}}\right)}^{m_{i}}.$$

If all SNGs receive their pro rata share based on a number of SNGs, then $m_{i}/q_{i}=1$ for each i, $\prod_{i=1}^{N}{\left(m_{i}/q_{i}\right)}^{m_{i}}=1$ and there is no revenue dispersion. Accordingly, the further is the mean from Equation (29) away from unity, the greater is revenue inequality across sets. Similarly, on the expenditure side, the geometric mean is:

(30) $$e^{I\left(s:q\right)}={\prod_{i=1}^{N}\left(\frac{s_{i}}{q_{i}}\right)}^{s_{i}}.$$

where $s={\left[s_{1},\ldots ,\;s_{N}\right]}^{\prime }$ and $q={\left[q_{1},\ldots ,\;q_{N}\right]}^{\prime }$ with $s_{i}$ and $q_{i}$ is the expenditure share and institutional share of the *i*th region.

### 4.4. A Numerical Example

One of the contributions of this paper is illustrated with a simple example. Consider two hypothetical nations V and L which exhibit the same degree of fiscal decentralisation, using typical measure of fiscal decentralisation index as discussed in [17]. It is now further assumed that these two countries consist of four subnational regions: A, B, C and D, each with different level of revenue (and expenditure). Table 1 provides data for this example. 

Column 2 shows that there is one small region in country V, region A. Revenue from region B is almost double that of D and forty times higher than that of region A. Columns 3 and 4 present the actual and average revenue shares for 4 regions in country V. By contrast, in country L, there is one large and three small regions. Region B accounts for more than 92 percent of the total revenue of all regions, and the remaining 8 percent is spread across the three small regions A, C, and D.

- Row 6 presents the standard deviations of the revenue shares of the two, 0.178 and 0.451. This clearly reveals that the distribution of revenue of country L is more dispersed than in V.
- Row 7 gives the values of the fiscal entropy, defined as where is the revenue share of SNG. The entropy value in country V is 0.484 and 0.146 in country L, as shown in columns 3 and 7 of row 8, respectively. If we were to assume alternatively that each region accounts for the same share of 25 percent, as shown by columns 4 and 8, there is no inequality, so that fiscal entropy for both countries is as in row 8, columns 4 and 8.
- Row 8 presents the fiscal inequality, the difference between the maximum level of the entropy, or 0.602, and the actual level. Fiscal inequality is 0.118 and 0.456 for countries V and L, respectively. Higher fiscal inequality in L means a greater degree of revenue dispersion among SNGs and, as a result, suggests a lower degree of fiscal decentralisation because revenue is allocated more disproportionately across regions.

To summarise this example, countries V and L may exhibit the same degree of fiscal decentralisation as discussed in [17]. But as there is much more fiscal inequality in country L, it can be reasonably concluded that the true situation may be different: there is less fiscally decentralised in country L. As such, studies on measuring various aspects of fiscal activities such as fiscal decentralisation should carefully take into account the dispersion of revenue (and expenditure) across regions. The entropic approach developed in this paper is able to accommodate these dispersions across subnational governments.

### 4.5. An Entropic Approach for Measuring Fiscal Decentralisation

We now turn to the application of this new framework into the Vietnamese context using its fiscal data across provinces and districts.

Table 2 presents the fiscal inequalities across subnational regions in Vietnam in 2015. The samples include 61 provinces and 5 major cities under direct management of the national government (Ha Noi, Ho Chi Minh City, Hai Phong, Da Nang and Can Tho) in Vietnam except for Binh Phuoc, and Ha Tinh provinces due to the unavailability of data. It is clear that within-province fiscal inequality accounts for 81.6 per cent and 93.6 per cent total inequality in terms of revenue and expenditure, respectively. This implies that the within-province fiscal inequality plays a more important role in total inequality of the distribution of revenue and expenditure across subnational regions in Vietnam. This is partly because each subnational region includes both provincial and local governments, and the provincial government is significantly larger than any local government within the same region. For example, for Ho Chi Minh City, the total share of 14.1 per cent in 2015, the state (city) government accounts for 9.9 per cent leaving only 4.2 per cent to be divided among the 24 local governments (districts) in Ho Chi Minh City. Another implication from the fiscal inequalities is that it is a more equality in an allocation of expenditure across subnational regions rather than that of revenue.

The above analyses indicate that, for revenue and expenditure, a within-province inequality accounts for 81.6 percent and 93.8 per cent, of the total fiscal inequality in Vietnam respectively. Overall, in Vietnam, within-province inequality appears to be a dominant factor. It is argued that a significantly higher percentage of the within-province inequality in total fiscal inequality, for both revenue and expenditure, in Vietnam demonstrates that dispersion of revenue raising capacity and expenditure responsibility across local governments is substantial. As such, it is the claim of this paper these dispersions should be incorporated into any measurement of a degree of fiscal decentralization for a particular country.

As a preliminary recommendation in the context of Vietnam, the simple average of the percentage of both within-province inequality from revenue and expenditure, to be named the average dispersions of revenue and expenditure (or DRE) which is 87.7 per cent, being the simple average of 81.6 per cent and 93.8 per cent, should be considered. Further tests should be conducted on the above analyses to ensure that any incorporation of the dispersion of revenue and expenditure across subnational governments in measuring the degree of fiscal decentralization is robust and truly reflects the relationship between the national government and the subnational governments in the allocation of revenue raising autonomy and expenditure responsibility. 

As an illustration for the case of Vietnam, in 2015, total revenue from subnational governments (including governments at the provincial and district levels) is 288,524 billion Vietnam Dong (VND). Total national government revenue in 2015 is VND998,217 billion. Total subnational government expenditure is VND1,033,973 billion and total national government expenditure is VND1,265,625 billion. A degree of fiscal decentralization for Vietnam is 0.22, being the ratio between total revenue from subnational governments and total revenue from both national and subnational governments, or 0.45, being the ratio between total subnational government expenditure and total government expenditure. One of these two ratios have been used in measuring fiscal decentralization in previous empirical analyses.

Until 2010, even with the most recently advanced index of fiscal decentralization, the IFD [17], the degree of fiscal decentralization in Vietnam is 0.35, being the geometric mean of the so-called “Fiscal Autonomy” of 0.28 (being the ratio between revenue and expenditure of subnational governments) and “Fiscal Importance” of 0.45 (being the ratio between total expenditure from subnational governments and total government (including subnational and national) expenditure. 

This paper considers that the dispersions of revenue raising capacity and expenditure responsibility are important and as such, these dispersions, derived from the framework of fiscal decentralization, should be incorporated into the final and true degree of fiscal decentralization of Vietnam. As a result, the true degree of fiscal decentralization in Vietnam is recommended to be approximately 0.31, which is the product between the IFD (0.45) and the DRE (87.7 per cent or 0.877).

## 5. Concluding Remarks

It has been widely considered that fiscal decentralization is an important aspect for a sustainable economic growth regardless of the current level of income across countries in the world. One of the difficult issues is to measure satisfactorily the degree of fiscal decentralisation across countries. In previous analyses, measurement of fiscal decentralisation in public finances has been very crude. Typically, either revenue or expenditure from subnational governments (“SNGs”) has been employed without taking into account the fiscal autonomy of lower level governments. Vo [17,18] developed the fiscal decentralisation index, the first of its kind, which accounts for both fiscal autonomy and fiscal importance of subnational governments. We argue that while Vo’s index is an advance on current practice, it is still not perfect as it assumes there is no dispersion of revenue and expenditure across regions. This index of fiscal decentralisation in relation to government revenue and expenditure are insensitive with the different distributions of revenue and expenditure among SNGs and a number of SNGs—the fundamental aspects for any country. In response to these potential limitations, an entropic approach to the analysis of subnational fiscal inequality has been developed in this paper. In response to this weakness, fiscal entropy and fiscal inequality measures are developed using information theory in this paper.

An application of the entropic approach developed in this paper for the case of Vietnam demonstrates that a true degree of fiscal decentralization of the country has effectively been reduced in comparison with other estimates. It is because Vietnam has experienced a high degree of fiscal dispersions across subnational governments in both raising revenue autonomy and expenditure responsibility between the national government and the provincial and district governments. As such, future academic studies on the issue of fiscal decentralization should consider the important aspect of fiscal dispersions across subnational governments in any measurement.

## Figures and Tables

**Table 1:** Illustrating Fiscal Inequality.

	Region/ Measures	Country V				Country L			
		Own-Sourced Revenue	Share in Total			Own-Sourced Revenue	Share in Total		
		($ millions)	Actual	Average	Difference	($ millions)	Actual	Average	Difference
	(1)	(2)	(3)	(4)	(5) = (4) − (3)	(6)	(7)	(8)	(9) = (8) − (7)
1.	A	3000	0.010	0.250	0.240	3300	0.011	0.250	0.239
2.	B	125,000	0.427	0.250	−0.177	271,390	0.926	0.250	−0.676
3.	C	97,000	0.331	0.250	−0.081	10,810	0.037	0.250	0.213
4.	D	68,000	0.232	0.250	0.018	7500	0.026	0.250	0.224
5.	Total	293,000	1.000	1.000	0.000	293,000	1.000	1.000	0.000
6.	Standard deviation		0.178	0.000			0.451	0.000	
7.	Entropy		0.484	0.602			0.146	0.602	
8.	Fiscal Inequality		0.118	0.000			0.456	0.000	

**Table 2:** Fiscal inequalities across subnational governments, Vietnam 2015.

Inequality Measure	Revenue	Expenditure
**Total Inequality**	0.762	0.625
Between-province inequality	0.140	0.039
Within-province inequality (WSI)	0.621	0.586
WSI as the percentage of total inequality	81.6	93.8
